# Difference of Intrahost Dynamics of the Second Human Pegivirus and Hepatitis C Virus in HPgV-2/HCV-Coinfected Patients

**DOI:** 10.3389/fcimb.2021.728415

**Published:** 2021-08-12

**Authors:** Yuanhao Liang, Fengyu Hu, Hang Fan, Linghua Li, Zhengwei Wan, Haiying Wang, Jingwei Shui, Yuanping Zhou, Yigang Tong, Weiping Cai, Shixing Tang

**Affiliations:** ^1^Department of Epidemiology, School of Public Health, Southern Medical University, Guangzhou, China; ^2^Guangzhou Eighth People’s Hospital, Guangzhou Medical University, Guangzhou, China; ^3^State Key Laboratory of Pathogen and Biosecurity, Beijing Institute of Microbiology and Epidemiology, Beijing, China; ^4^Department of Infectious Diseases, Nanfang Hospital, Southern Medical University, Guangzhou, China; ^5^School of Life Science and Technology, Beijing University of Chemical Technology, Beijing, China; ^6^Wenzhou Institute, University of Chinese Academy of Sciences, Wenzhou, China

**Keywords:** HPgV-2, HCV, HCV/HPgV-2 coinfection, intrahost variants, cytokine

## Abstract

**Background:**

The second human pegivirus (HPgV-2) and hepatitis C virus (HCV) belong to the Flaviviridae family and share some common genome features. However, the two viruses exhibit significantly different genetic diversity. The comparison of intrahost dynamics of HPgV-2 and HCV that mainly reflect virus-host interactions is needed to elucidate their intrahost difference of genetic diversity and the possible mechanisms.

**Methods:**

Intrahost single nucleotide variations (iSNVs) were identified by means of next-generation sequencing from both cross-sectional and longitudinal samples from HPgV-2- and HCV-coinfected patients. The levels of human cytokines were quantified in the patient before and after HCV elimination by the treatment of direct-acting antivirals (DAA).

**Results:**

Unlike HCV, the viral sequences of HPgV-2 are highly conserved among HPgV-2-infected patients. However, iSNV analysis confirmed the intrahost variation or quasispecies of HPgV-2. Almost all iSNVs of HPgV-2 did not accumulate or transmit within host over time, which may explain the highly conserved HPgV-2 consensus sequence. Intrahost variation of HPgV-2 mainly causes nucleotide transition in particular at the 3rd codon position and synonymous substitutions, indicating purifying or negative selection posed by host immune system. Cytokine data further indicate that HPgV-2 infection alone may not efficiently stimulate innate immune responses since proinflammatory cytokine expression dramatically decreased with elimination of HCV.

**Conclusion:**

This study provided new insights into the intrahost genomic variations and evolutionary dynamics of HPgV-2 as well as the impact of host immune selection and virus polymerase on virus evolution. The different genetic diversity of HPgV-2 and HCV makes HPgV-2 a potential new model to investigate RNA virus diversity and the mechanism of viral polymerase in modulating virus replication.

## Introduction

The second human pegivirus (HPgV-2), a single-stranded positive-sense RNA virus was first reported in 2015 and belongs to the family of Flaviviridae (Berg et al., 2015; Kapoor et al., 2015). Since then, HPgV-2 has been documented in the USA, UK, China, Vietnam, Cameroon, Iran, etc. (Bonsall et al., 2016; Anh et al., 2018; Bijvand et al., 2018; Wang et al., 2018a; Rodgers et al., 2019). It is noteworthy that HPgV-2 is epidemiologically tightly associated with HCV infection (Berg et al., 2015; Anh et al., 2018; Kandathil et al., 2018; Wang et al., 2018a; Wang et al., 2018b; Rodgers et al., 2019) and shares several common genomic features with HCV (Kapoor et al., 2015; Bonsall et al., 2016; Coller et al., 2016). HCV is a genetically diverse RNA virus and has seven genotypes and 67 subtypes (Smith et al., 2014) due to high error rate of RNA polymerase during replication (Moradpour et al., 2007). In contrast, the overall nucleotide identity among HPgV-2 consensus sequences worldwide is about 94%, and no apparent hypervariable regions are observed within HPgV-2 genome (Berg et al., 2015; Wang et al., 2018b; Rodgers et al., 2019; Sridhar et al., 2019; Forberg et al., 2020). These results indicate the significant difference of HPgV-2 and HCV with respect to their interhost genetic diversity. However, viral consensus sequences may disguise the true intrahost variation spectra of closely related viral genomes (Shaw et al., 2020). Therefore, it is needed to compare intrahost dynamics of HPgV-2 and HCV that mainly reflect virus-host interactions to elucidate their intrahost difference of genetic diversity.

Within host, virus evolution experiences mutation and immune selection to form genetic variants with various levels of fitness. HCV, for example, usually composes of a large number of closely related but distinct viral sequences, which is referred as quasispecies (Ramachandran et al., 2011), indicating great intrahost variation of HCV genome in particular the hypervariable region (HVR) of HCV at N-terminal of the structural protein E2 (Brambilla et al., 1998). However, the intrahost variation of HPgV-2 is still unknown, which in turn impedes assessment of HPgV-2 genetic variability and evolution dynamics. Forberg et al. reported that HPgV-2 remains highly stable in one patient over 7 weeks compared with HCV (Forberg et al., 2020). Due to coinfection of HPgV-2 and HCV, it is possible to compare the intrahost diversity of both HPgV-2 and HCV in parallel in HPgV-2/HCV-coinfected patients by means of deep sequencing of viral genomes from serial clinical samples to shed light on viral evolutionary dynamics, human-to-human transmission, and the size of transmission bottleneck (Gire et al., 2014; Chen et al., 2018; Shen et al., 2020). In our study, longitudinal samples from HCV/HPgV-2-coinfected patients were collected to analyze the dynamics of intrahost single nucleotide variations (iSNVs) and to compare the diversity rate of HPgV-2 and HCV. Furthermore, expression levels of human cytokines were analyzed to investigate the role of HPgV-2 and HCV in regulating innate immune responses in the patients before and after HCV elimination by the treatment of direct acting antivirals (DAAs).

## Materials and Methods

### Patients and Sample Collection

A total of eight patients diagnosed with coinfection of HCV and HPgV-2 in Nanfang Hospital and Guangzhou Eighth People’s Hospital were included in our study, and eleven plasma samples were collected at different time points (**Supplementary Table 1**). All the plasma samples were aliquoted and stored at −80°C until analysis. All the participants enrolled in the study provided written informed consent. This study has been approved by the ethical committee of Nanfang Hospital (NFEC-2017-046) and Guangzhou Eighth People’s Hospital (No. 201816107) according to the Declaration of Helsinki.

### Next-Generation Sequencing Analysis of HPgV-2 and HCV

Viral RNAs were extracted from the plasma samples of HCV/HPgV-2-coinfected patients using High Pure Viral RNA Kit (Roche Diagnostics, Indianapolis, USA) and reverse transcribed into cDNA using Transcriptor First Strand cDNA Synthesis Kit (Roche Diagnostics, Indianapolis, USA). Twelve pairs of HPgV-2-specific primers with five nucleotides overlap used to amplify six fragments covering the near full-length genome (NFLG) of HPgV-2 have been described previously (Wang et al., 2018b). Additionally, two sets of HCV-specific primer pairs for genotypes 3a (primer set 1) and 6a (set 2) were designed to amplify HCV HVR1 (**Supplementary Table 2**). PCR products were purified by using Universal DNA Purification Kit (Tiangen Biotech, Beijing, China) according to the manufacturer’s instructions. The pooled PCR products were prepared with the multiplex NGS library using a Nextera XT Sample Preparation Kit (Illumina, San Diego, CA, USA). The Illumina MiSeq platform was used to generate 2 × 150 bp paired-end reads. Sequencing data of the current study were deposited at the Sequence Read Archive (SRA) database of the National Institutes of Health under the accession number PRJNA743193. Quality control and error correction were implemented according to the amplicon-sequencing error patterns generated by Illumina’s MiSeq and Nextera XT Sample Preparation Kit (Schirmer et al., 2015). Bayes Hammer implemented in SPAdes v3.5.0 was used for error correction (Nikolenko et al., 2013). Reads without corresponding paired reads were disregarded.

### Detection of Intrahost Variants

The clean reads were aligned with the reference genome of HPgV-2 (accession no. KT427414) and HCV genotype 3a (accession no. D17763) or 6a (accession no. KC844037), respectively, using CLC genomics Workbench version 9.0 (Qiagen, Hilden, Germany). The calling process of iSNVs is as follows: for each nucleoside of HPgV-2 or HCV genome, the aligned low-quality bases (*Q* < 20) and indels were excluded to reduce the possible false positivity; then, a series of criteria were used to call iSNVs including (1) minor allele frequency of ≥5%; (2) depth of the minor allele of ≥5; and (3) strand bias of the minor allele of <10-fold; and (4) the variation sites outside of the amplification primers. The allele frequency for different nucleotide substitution was the number of substitutions divided by the total number of nucleotides at each specific site among the reads obtained from sequencing. The mean allele frequency for different substitutions was the average of the values from eight patients analyzed. To define the nucleotide polymorphism of HPgV-2 and HCV at the population level, 22 HPgV-2 sequences and 26 HCV sequences were downloaded from GenBank, respectively (**Supplementary Tables 3, 4**).

### Nucleotide Diversity Analysis

The average number of nucleotide difference per site (pi value) was calculated by using DnaSP v5.1 (Librado and Rozas, 2009). Pi value for both HPgV-2 and HCV sequences was estimated by a sliding window method with window length of 200 nucleotides and step of 25 nucleotides. Pi value for each window was calculated and assigned to the nucleotide position at the midpoint of each overlapping window. Furthermore, MEGA7 was used to calculate the pair-wise nucleotide difference and the genetic distance, which was corrected by Jukes and Cantor’s method. Diversity rate was estimated as the number of nucleotide differences between two sequences divided by the time period observed and presented as substitution/site/year.

### Cytokine Profile Analysis

The relative expression levels of 640 human cytokines were analyzed by the G-Series Human Cytokine Antibody Array 640 (GSH-CAA-640, Raybiotech, Guangzhou, China). The array slides were treated and processed according to manufacturer’s instructions. The raw data were processed by removing background and normalizing the differences between array detections. Differentially expressed proteins were defined as those with absolute change of ≥2-fold for HPgV-2/HCV-coinfected patient before and after DAA therapy to eliminate HCV, or ≥-4-fold when comparing between HPgV-2/HCV-coinfected patient and HCV mono-infected patient, as well as between HPgV-2/HCV-coinfected patient after HCV elimination and healthy blood donor.

### Statistical Analysis

Data were analyzed using R software, version 3.5.2 (R Foundation for Statistical Computing). Categorical variables were expressed as counts and percentages and compared using Chi-square analysis or Fisher’s exact test as appropriate whereas allele frequency values were summarized as the median and interquartile range (IQR) and compared using Wilcoxon rank-sum test.

## Results

### Intrahost Quasi-Species and Less Interhost Diversity of HPgV-2 Genome

We first analyzed the number of iSNVs and normalized them by the genome length as #iSNV/kb to represent the rate of intrahost viral diversity. Previous studies indicated high conservation of HPgV-2 consensus sequences worldwide (Forberg et al., 2020). In our study, five NFLGs of HPgV-2 from four subjects were constructed. The number of iSNVs is quite different among HPgV-2-infected subjects and ranges from 18.0 to 237.0 with a mean of 124.8 ± 85.2 whereas the #iSNV/kb ranges from 1.9 to 24.9 with an average of 13.2 ± 9.0 (**Table 1** and **Supplementary Table 5**). Since HCV HVR1 is frequently used for assessing HCV diversity, we also analyzed #iSNV/kb in HCV HVR1, which is 30.77–69.23 with an average of 52.3 ± 16.0 among the sequences obtained from three subjects in the first time point (**Table 1**). Interestingly, patients No. 1563 and No. C346 showed relatively high frequency of HPgV-2 iSNVs/kb, which was even close to that of HCV HVR1 (**Table 1**). Our results revealed similar quasi-species of HPgV-2 as HCV but much small interhost difference of genetic diversity of HPgV-2 genome.

**Table 1:** Distribution of intrahost single nucleotide variations (#iSNVs/kb) in the genome of HPgV-2 and HCV HVR1.

Viral genome	HCV121^†^		NO.19^†^		NO.64^†^		NO.655	NO.1241	NO.1563	C346	Jx18052
	t0	t44	t0	t216	t0	t36					
**HPgV-2**											
Whole	18 (1.90)	20 (2.11)	172 (18.14)	–	–	–	–	–	–	237 (25.00)	72 (7.56)
5′-UTR	0 (0)	0 (0)	0 (0)	2 (6.12)	0 (0)	1 (3.06)	1 (3.06)	3 (9.17)	1 (3.06)	0 (0)	1 (3.06)
S	0 (0)	0 (0)	1 (4.24)	0 (0)	0 (0)	0 (0)	2 (8.48)	3 (12.72)	0 (0)	5 (21.20)	0 (0)
E1	2 (3.49)	0 (0)	12 (20.94)	1 (1.75)	1 (1.75)	0 (0)	3 (5.24)	12 (20.94)	0 (0)	18 (31.41)	1 (1.75)
E2	4 (3.77)	4 (3.77)	33 (31.10)	1 (0.94)	5 (4.71)	0 (0)	4 (3.77)	20 (18.85)	8 (7.54)	40 (37.7)	17 (16.02)
X	2 (2.81)	2 (2.81)	14 (19.67)	NT	1 (1.41)	NT	6 (8.43)	9 (12.65)	10 (14.05)	35 (49.18)	9 (12.65)
NS2	2 (2.78)	0 (0)	5 (6.95)	NT	2 (2.78)	NT	NT	NT	NT	11 (15.29)	4 (5.56)
NS3	1 (0.53)	2 (1.06)	21 (11.13)	NT	3 (1.59)	NT	0 (0)	26 (13.78)	7 (3.71)	20 (10.60)	13 (6.89)
NS4	0 (0)	2 (2.20)	19 (20.90)	NT	1 (1.10)	NT	1 (1.10)	21 (23.10)	28 (30.80)	18 (19.80)	7 (7.70)
NS5A	3 (2.18)	5 (3.63)	43 (31.25)	NT	NT	NT	1 (0.73)	10 (7.27)	88 (63.98)	62 (45.05)	7 (5.09)
NS5B	4 (2.35)	5 (2.94)	26 (15.28)	NT	NT	NT	NT	0 (0)	NT	29 (17.04)	13 (7.64)
3′-UTR	NT	NT	NT	NT	NT	NT	NT	NT	NT	NT	NT
**HCV**											
HVR1	15 (57.03)	29 (110.27)	8 (30.77)	16 (61.54)	18 (69.23)	23 (88.46)	NT	NT	NT	NT	NT

HVR1, hypervariable region 1; NT, not tested; #iSNVs/kb, the number of iSNVs called per kb.

^†^Longitudinal samples were available from three HCV/HPgV-2-coinfected subjects: HCV121 (collected 44 weeks apart), NO.19 (collected 216 weeks apart), and NO.64 (collected 36 weeks apart).

**Figure 1:**
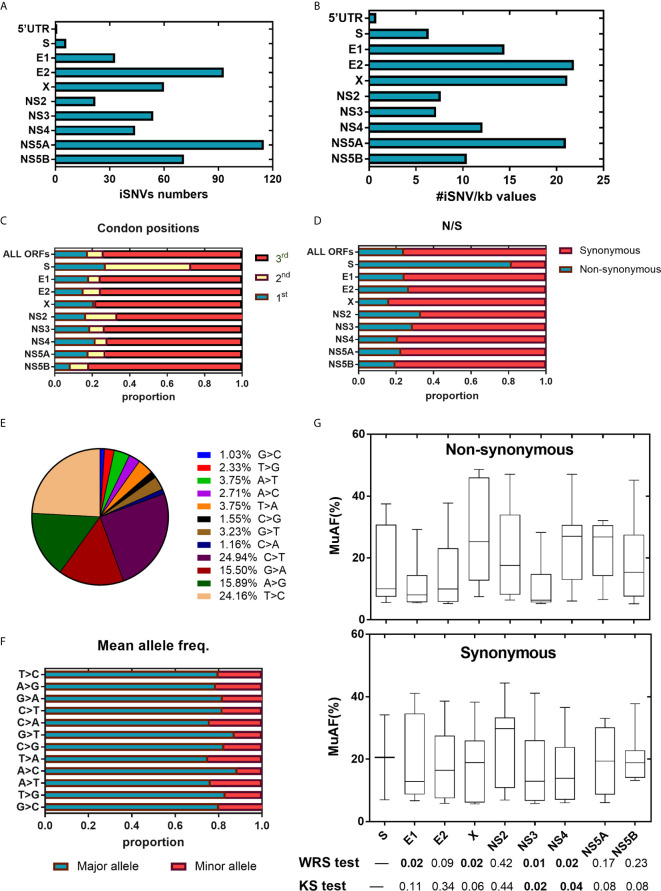
HPgV-2 iSNV distributions along the viral genome. **(A)** Total iSNV numbers along HPgV-2 genomic regions and ORF regions. **(B)** The #iSNVs/kb values along HPgV-2 genomic regions and ORF regions. **(C)** Distribution of iSNVs at codon positions by gene. **(D)** Distribution of nonsynonymous (N)/synonymous (S) iSNVs by gene. **(E)** Proportion of nucleotide substitutions among all iSNV sites were shown in pie chart. **(F)** Mean allele frequency of each nucleotide substitution, T>C indicated T change to **(C, G)** Box plots show mutated allele frequency (MuAFs) for nonsynonymous and synonymous iSNVs of HPgV-2 ORFs. Boxes represent the interquartile range (IQR) between the first and third quartiles; horizontal lines inside the boxes indicate the median. Statistical tests were performed between nonsynonymous and synonymous iSNVs of each ORF. WRS test, Wilcoxon rank-sum test; KS test, Kolmogorov-Smirnov test. **(A, B)** presented data from four patients which have been sequenced the near whole HPgV-2 genome whereas **(C–G)** presented data from all eight patients.

**Figure 2:**
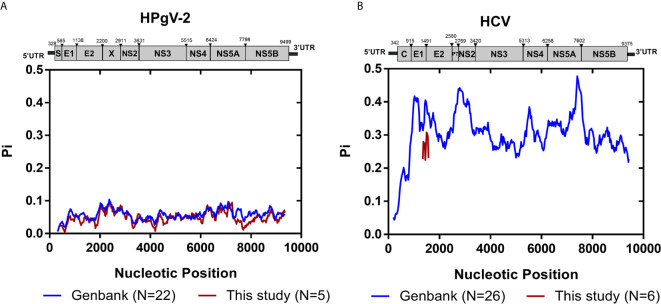
Genomic polymorphism of HPgV-2 and HCV. **(A)** Genomic polymorphism among 22 strains of HPgV-2 obtained from the GenBank database and five HPgV-2 consensus sequences of this study are respectively shown. The nucleotide positions are numbered according to the human pegivirus 2 isolate UC0125 (GenBank accession number KT427414). **(B)** Genomic polymorphism among 26 strains of HCV obtained from the GenBank database and six HCV HVR1 consensus sequences of this study are respectively shown. The nucleotide positions are numbered according to HCV strain H77 (GenBank accession number NC_004102). Graph was constructed using sliding window of 200 nucleotides and steps of 25 nucleotides.

We then examined the distribution of iSNVs along the HPgV-2 genome. In our study, five NFLGs of HPgV-2 with 5′-UTR sequences were constructed and cover 95% of the entire genome. As expected, 99.8% (498/499) of iSNVs accumulate in the coding regions (**Figure 1A**). Although at the population level, there are no significant hypervariable genes of HPgV-2 (**Figure 2A**) compared with HCV (**Figure 2B**), we observed the relatively high variable genes in HPgV-2 E1, E2, X, and NS5A. The corresponding #iSNV/kb values are 14.4 ± 12.4, 22.2 ± 13.2, 21.1 ± 17.3, and 20.9 ± 18.0, respectively (**Figure 1B**). The intrahost genetic diversity of HPgV-2 (**Figure 1B**) is consistent with the interhost genetic polymorphism observed at the population level of HPgV-2 genomes (**Figure 2A**). Of note, no substitution was identified in HPgV-2 5′-UTR, which acts as a regulatory region of viral translation initiation, indicating its critical role in regulating virus replication.

We also compared the genetic diversity of HPgV-2 and HCV by analyzing the evolution rate in HCV HVR1 and the equivalent E1/2 gene of HPgV-2. We found that the genome diversity rate ranges from <0.01 to 0.56 × 10^−2^ substitution per site per year for HPgV-2, and 0.71–12.03 × 10^−2^ substitution per site per year for HCV, respectively, indicating 2.29–932-fold difference between HCV and HPgV-2 (**Table 2**). The difference is consistent with the observation at the population level of whole viral genome. Furthermore, there was no significant association between viral load and the number of iSNVs for both HPgV-2 (Spearman’s rho = 0.800, *p* = 0.200) or HCV (Spearman’s rho = −0.632, *p* = 0.368).

**Table 2:** Diversity rate of HPgV-2 and HCV from HCV/HPgV-2-coinfected patients.

	Sample ID	Genetic distance (substitution/site)	Time intervals (years)	Diversity rate (substitution/site/year)
HPgV-2				
nt20-2199**^†^**	HCV121	<0.0001	0.92	<0.0001
nt20-2199**^†^**	NO.19	0.0138	4.50	0.0031
nt20-2199**^†^**	NO.64	0.0042	0.75	0.0056
HCV HVR1				
nt1321-1583**^¶^**	HCV121	0.0854	0.92	0.0932
nt1278-1537**^§^**	NO.19	0.0318	4.50	0.0071
nt1278-1537**^§^**	NO.64	0.0903	0.75	0.1203

^†^Nucleotides are numbered according to the human pegivirus 2 isolate UC0125 (GenBank accession number KT427414).

^¶^Nucleotides are numbered according to the HCV subtype 3a isolate NZL1 (GenBank accession number D17763).

^§^Nucleotides are numbered according to the HCV subtype 6a isolate ZS221 (GenBank accession number KC844037).

### Purifying Negative Selection in HPgV-2 and Positive Selection in HCV

We then investigated the distribution of iSNVs within each open reading frame (ORF) to reflect the selection pressure posed by host immune system upon virus infection. In general, majority (74.0%, 568/768) of the iSNVs occur at the 3rd codon position in most HPgV-2 ORFs (**Table 3** and **Figure 1C**) and result in synonymous substitutions, suggesting a purifying or negative selection from host immune system (**Figure 1D**). An exception was observed in HPgV-2 S gene, in which significantly more iSNVs locate in the 1st and 2nd codon positions rather than the 3rd codon position (**Figure 1C**) while the ratio of nonsynonymous over synonymous substitutions (N/S) for HPgV-2 S gene is much bigger than the whole genome of HPgV-2 (4.5 *vs.* 0.3, **Figure 1D**). These results suggest that HPgV-2 S gene may undergo more positive selection than the other genes and may be under less host selection pressure although its expression and function remain to be determined (Berg et al., 2015; Kapoor et al., 2015). Furthermore, we found that the distribution of iSNVs at each codon position is quite different between HPgV-2 and HCV (**Table 3**, *p* = 0.0020) in particular in the 1st (17.7% *vs.* 39.0%) and the 3rd (74.0% *vs.* 48.8%) codon positions (**Table 3**). The ratio of N/S is much smaller for HPgV-2 than HCV (0.32 *vs.* 1.41, *p* < 0.001, **Table 3**), suggesting purifying and negative selection rather than positive selection posed on HPgV-2 infection.

**Table 3:** Characteristic of intrahost single nucleotide variations in the genome of HPgV-2 and HCV HVR1.

Characteristic	HPgV-2	HCV	*p-*Value
**Distribution at codon positions**			
1st	136 (17.7%)	16 (39.0%)	0.0020*
2nd	64 (8.3%)	5 (12.2%)	
3rd	568 (74.0%)	20 (48.8%)	
**Type of amino acid substitution**			
Synonymous	582 (75.8%)	17 (41.5%)	<0.0001*
Nonsynonymous	186 (24.2%)	24 (58.5%)	
Ratio of N/S	0.32	1.41	
**Type of nucleotide substitution**			
Transitions	623 (80.5%)	30 (73.2%)	0.3132
Transversions	151 (19.5%)	11 (26.8%)	
**Mean allele frequency (%)**			
Major allele	83.8 (71.5–92.1)	86.5 (84.8–89.9)	0.1800^†^
Minor allele	16.2 (7.9–28.5)	13.5 (10.1–15.2)	0.1800^†^
**Mean mutated allele frequency (%)**			
Synonymous	16.4 (8.1–28.5)	13.6 (10.4–15.6)	0.5900^†^
Nonsynonymous	15.6 (6.7–28.8)	13.4 (9.6–14.1)	0.2470^†^

*Categorical variables were expressed as counts and percentages and compared using Chi-square analysis or Fisher’s exact test.

^†^The frequency values expressed as median (interquartile range). The value of major allele, minor allele, synonymous mutated allele, and nonsynonymous mutated allele between HPgV-2 and HCV are compared with each other with the Wilcoxon rank-sum test.

In addition to codon position, we further examined the type of nucleotide substitution and allele composition caused by HPgV-2 iSNVs. All iSNV sites are examined as a mixture of only two nucleotides. We found that 80.5% of HPgV-2 iSNV result in nucleotide transition rather than transversion. Among them, 49.1% of the total substitutions are C/T or T/C substitution and 31.4% are A/G or G/A substitution (**Figure 1E**). The major allele frequency is 83.8% (71.5%–92.1%) (**Table 3**; **Figure 1F**). Since the distribution of mutated allele frequency (MuAF) would also reflect host selection (Boyko et al., 2008; Li et al., 2010; Acevedo et al., 2014), we plotted the MuAF spectra by HPgV-2 viral gene (**Figure 1G**). In general, except for X gene and NS4 gene, the nonsynonymous iSNVs in the other coding regions show larger proportion of low-frequency alleles (**Figure 1G**), implying strong purifying selection for the nonsynonymous iSNVs. However, in X gene and NS4 gene, the MuAF for nonsynonymous iSNVs was significantly higher than its synonymous iSNVs (25.3% *vs.* 18.9%, *p* = 0.02 for X gene; 27.0% *vs.* 13.9%, *p* = 0.02 for NS4 gene). Of note, HPgV-2 NS3 gene appears to be highly conserved because the difference of the MuAF distribution of nonsynonymous iSNVs is significantly smaller than that of its synonymous counterpart (*p* < 0.05, **Figure 1G**), suggesting its important role as viral protease to maintain virus replication. In contrast, among the 41 HCV iSNVs identified, 58.5% (24/41) of them are nonsynonymous substitution (**Table 3**; **Supplementary Table 6**; **Supplementary Figures 1A, D**), indicating a positive selection. Interestingly, there is no significant difference between HPgV-2 and HCV with respect to the type of iSNV-related nucleotide substitution (transition or transversion), mean allele frequency, and mean mutated allele frequency (**Table 3**), indicating their shared common features.

### Significantly Less Co-Occurring iSNVs in HPgV-2 Than in HCV

**Figure 3:**
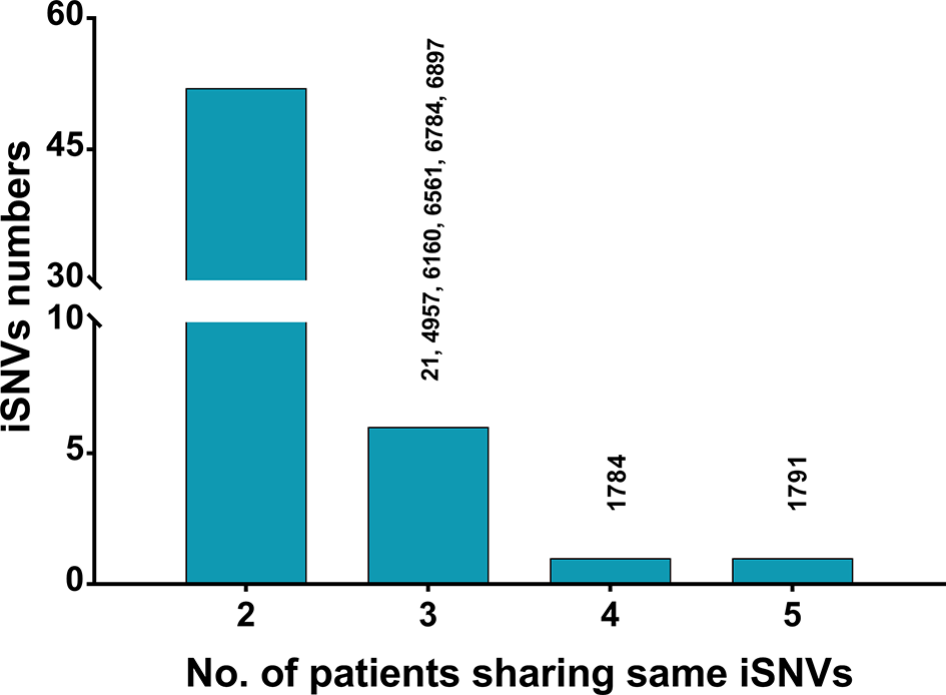
Statistics of shared HPgV-2 iSNV sites among patients. Each bar represents the number of iSNV sites occurring in a given number of patients (≥2). Sites shared in ≥3 samples are shown on top of the bars.

The co-occurring iSNVs among patients could be used to estimate transmission bottleneck and human-to-human transmission (Gire et al., 2014; Emmett et al., 2015; Park et al., 2015). For HPgV-2, 92.5% (739/799) of the iSNVs occurred in only one subject whereas 6.5% (52/799) and 1.0% (8/799) of the iSNVs were shared by two patients and more than three patients, respectively (**Figure 3**). Furthermore, no co-occurring iSNVs were found in two out of three HPgV-2-infected subjects whereas only 11.1% (2/18) of the iSNVs were shared in one patient at different time points over the course of HPgV-2 infection (**Table 4**). In addition, the shared HPgV-2 iSNVs did not result in the change of the major alleles over time. In contrast, for HCV HVR1, the frequency of co-occurring HCV iSNVs ranges from 37.5% to 88.9% over time in the three patients analyzed, and 87.5% (28/32) of the shared iSNVs resulted in the transfer from minor alleles to major alleles (**Table 4**). Of note, among the subjects analyzed, no shared iSNVs were identified in the HPgV-2 E2 gene fragment, which is equivalent to HCV HVR1 (**Table 4**).

**Table 4:** Shared intrahost single nucleotide variations between the longitudinal samples of HCV/HPgV-2-coinfected patients.

Sample ID	Gene analyzed	No. of iSNV (%)			
		Time point T1	Time point T2	Shared for T1/T2	Minor to major allele transfer
HCV121	HPgV-2 NFLG nt20-9400**^†^**	18	20	2 (2/18, 11.1%)	0
	HPgV-2 E2 nt2000-2199**^†^**	0	0	0	0
	HCV HVR1 nt1321-1583**^¶^**	15	28	13 (13/15, 86.7%)	12 (12/13, 92.3%)
No. 19	HPgV-2 NFLG nt20-2199**^†^**	45	4	0	0
	HPgV-2 E2 nt2000-2199**^†^**	7	0	0	0
	HCV HVR1 nt1321-1583**^§^**	8	16	3 (3/8, 37.5%)	0
No. 64	HPgV-2 NFLG nt20-2199**^†^**	6	1	0	0
	HPgV-2 E2 nt2000-2199**^†^**	0	0	0	0
	HCV HVR1 nt1321-1583**^§^**	18	23	16 (16/18, 88.9%)	16 (16/16, 100.0%)

^†^Nucleotides are numbered according to the human pegivirus 2 isolate UC0125 (GenBank accession number KT427414).

^¶^Nucleotides are numbered according to the HCV subtype 3a isolate NZL1 (GenBank accession number D17763).

^§^Nucleotides are numbered according to the HCV subtype 6a isolate ZS221 (GenBank accession number KC844037).

### Downregulation of the Innate Immune After Elimination of HCV, But Not HPgV-2

**Figure 4:**
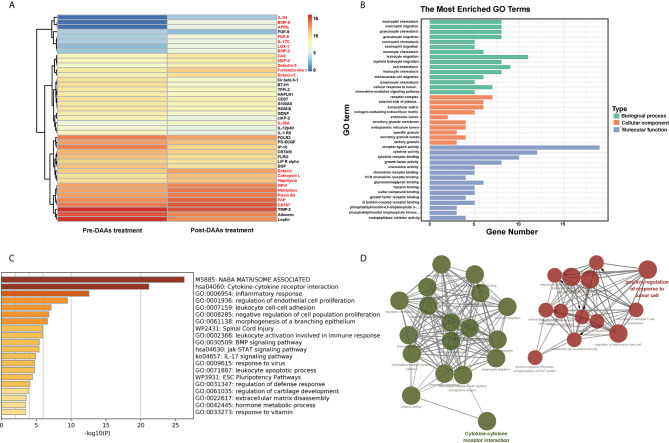
Pathway analysis of 43 differentially expressed proteins in HPgV-2/HCV-coinfected patients between pre-DAAs treatment and post-DAA therapy. **(A)** Heatmap plots showing differentially expressed human cytokines, 21 upregulated cytokines labeled in red font while 22 downregulated cytokines labeled in black font. **(B)** The Gene Ontology enrichment analysis by “clusterProfiler” package in R software, the top 15 terms of biological process (BP), cellular component (CC), and molecular function (MF) are shown in bar chart. **(C)** The Gene Ontology processes enriched by Metascape platform and the top 20 terms are shown in bar chart. **(D)** The interaction networks of biological process using Cytoscape plug-in ClueGO show that the 43 differentially expressed cytokines mainly enriched in two function clusters including cytokine-cytokine receptor interaction and positive regulation of response to tumor cell.

DAA therapy to eliminate HCV can reduce innate immune response and was adapted to examine if HPgV-2 is associated with innate immune activation. Patient HCV121 was coinfected with HPgV-2 and HCV and was treated with DAA (Wan et al., 2020) (**Supplementary Table 7**). After DAA treatment for 3 months, HCV RNA was undetectable while HPgV-2 RNA remained positive, but HPgV-2 viral load decreased by 10-fold (**Supplementary Table 7**). We tested plasma samples collected before and after DAA treatment and identified 43 differentially expressed cytokines with more than twofold difference including 21 upregulated and 22 downregulated cytokines (**Figure 4A** and **Supplementary Table 8**). We observe a resetting of innate immune antiviral responses and an increase in adaptive immune responses in the peripheral blood, such as the significant decreases in IL-1 and chemokine CXCL10 and reduced expression of the innate immune signaling molecules (**Figure 4A**). Further Gene Ontology enrichment analysis of these differentially expressed cytokines revealed that their molecular functions mainly relate to cytokine and chemokine activity as well as receptor binding and receptor ligand activity (**Figure 4B**). Their biological processes include migration and chemotaxis of leukocyte, granulocyte, neutrophil, etc. (**Figure 4B**). These results are supported by gene ontology processes enriched by Metascape platform (**Figure 4C**). The interaction network analysis indicates that most upregulated cytokines are responsible for modulating activated T-cell proliferation and natural killer cell-mediated immune response while the downregulated cytokines mainly affecting the migration of inflammatory cells and chemotaxis (**Figure 4D**). Of note, after HCV elimination by DAA and under HPgV-2 mono-infection, proinflammatory cytokines including IL-12p40, IP-10, Ck beta 8-1, and IL-1 R5 were significantly downregulated while cytokines to regulate innate immune response such as Toll-like receptors, interferon, and interleukin-1 remained unchanged. A previous study has observed significant downregulation of innate immune regulators in HCV-infected patients following DAA treatment (Burchill et al., 2017). Moreover, analysis of differentially expressed cytokine profile between HPgV-2/HCV-coinfected patient and HCV mono-infected patient indicated that the 32 differentially expressed cytokines mainly contributed to cytokine-cytokine receptor interaction and positive regulation of cytokine production as well as intercellular signaling, in particular in the positive regulation of nitric-oxide synthase biosynthetic process, neutrophil chemotaxis, cell-cell adhesion mediator activity, and cytokine-cytokine receptor interaction (**Supplementary Figures 2A, B**). However, there are no difference with regard with the expression of IL-12p40, IP-10, Ck beta 8-1, and IL-1 R5 (**Supplementary Table 10**). Furthermore, a comparison of differentially expressed cytokine profile between HPgV-2/HCV-coinfected patient with elimination of HCV and healthy blood donor suggested that after HCV elimination by DAA and under HPgV-2 mono-infection, the expression of proinflammatory cytokines such as IL-12p40, IP-10, Ck beta 8-1, and IL-1 R5 were downregulated to a level similar to the healthy control (**Supplementary Table 10** and **Supplementary Figures 2C, D**). Therefore, our results suggest that HPgV-2 infection alone may not stimulate robust adaptive and strong innate immune response.

## Discussion

Lack of proofreading activity of viral polymerase and rapid evolution result in high mutation rate of RNA viruses, which are characteristic of swarms of variants to escape from host immune surveillance (Steinhauer et al., 1992). Virus variants can be classified into different genotypes or just exist as quasi-species, i.e., a group of viruses bearing genome-wide distributed mutations (Vignuzzi et al., 2006). HCV is highly mutated RNA virus with seven genotypes, 67 confirmed subtypes, and 20 provisionally assigned subtypes (Smith et al., 2014). Interestingly, HCV and HPgV-2 are closely related. They not only always exist as coinfection but also share some common viral genome features (Kapoor et al., 2015; Bonsall et al., 2016; Coller et al., 2016). However, unlike HCV, HPgV-2 sequences are far more conserved and no viral genotypes have been identified until now (Forberg et al., 2020). All the knowledge about genetic diversity of HPgV-2 comes from the comparison of the consensus sequences of HPgV-2 from different subjects worldwide and represents the interhost features of viral genome. It is necessary to characterize the intrahost features of HPgV-2 and to provide molecular evidence to explain the much less diversity of HPgV-2 sequences observed at the population level. In the current study, we analyzed the distribution of iSNVs along HPgV-2 genome in the longitudinal patient samples using NGS. Several important findings include: (1) much lower evolution rate of HPgV-2 than HCV, which is consistent with previous findings at population level and further confirms the feature of less prone mutation of HPgV-2; (2) much less iSNVs and co-occurring iSNVs in HPgV-2 than in HCV, and more synonymous than nonsynonymous substitutions in HPgV-2 than in HCV. These results reveal low intrahost variation under purifying selection of HPgV-2; (3) quasi-species of HPgV-2 and significant intrahost difference among HPgV-2-infected subjects, suggesting multiple mechanisms and factors to regulate HPgV-2 replication.

For the first time, our study indicates that like other RNA viruses, HPgV-2 exhibits intrahost variation and quasi-species although the level of quasi-species is generally lower in HPgV-2 than in HCV. Furthermore, intrahost mutation of HPgV-2 could be very high in some subjects. Our results provide evidence to support the RNA-dependent RNA polymerase (RdRp) of HPgV-2 as an error-prone polymerase similar to HCV RdRp (Forberg et al., 2020). Therefore, the conservation of HPgV-2 genome may not be due to the different activity of HPgV-2 RdRp. One explanation for the highly identical genome sequences observed in HPgV-2 could be because of lower error rate during virus replication and neutral or purifying selection from host immune system because majority of the iSNVs identified in HPgV-2 occur at the 3rd codon position and are associated with nucleotide transition and synonymous substitutions (**Table 1** and **Figures 1C, D**). We also observed that in the HPgV-2/HCV-coinfected patient, DAA treatment successfully eliminated HCV and could significantly downregulate innate immune response. Although the viral load of HgV-2 also decreased by 10-fold, our results suggest that DAA cannot eliminate HPgV-2 or the efficient replication of HPgV-2 may need the help of HCV. These results suggest that HPgV-2 per se does not stimulate strong innate immune response to pose high selection pressure upon HPgV-2 infection. Our results are in line with our previous findings that HPgV-2 infection results in limited or absence of pathogenicity (Wang et al., 2018b; Sridhar et al., 2019) and is at least not associated with viral hepatitis although it always coinfects with HCV (Wang et al., 2018b; Wan et al., 2020). Therefore, for HPgV-2/HCV-coinfected subjects, even though HPgV-2 confronts strong immune selective pressure, the immune responses may be mainly induced by HCV while the specific immune response against HPgV-2 may be moderate, which is in accordance with the decreased pro-inflammatory cytokines expression after HCV elimination (**Figure 4A**). The unique feature may favor HPgV-2 replication without the need of rapidly mutating and escaping immune surveillance. Another possibility is that HPgV-2 may adapt other fidelity-modulating mechanisms to maintain its fidelity to fulfill virus viability and fitness during virus replication. We know that the RdRp encoded by RNA viruses is typically not assisted by proofreading and may need to independently control its replication fidelity. Liu et al. reported that the classical swine fever virus (CSFV) adapts a unique intramolecular fidelity-modulating mechanism in which the fidelity of RdRp highly relies on the correct intramolecular interactions between the N-terminal domain (NTD) of CSFV NS5B protein and the RdRP palm domain (Liu et al., 2018). Since both HPgV-2 and CSFV belong to the same family of Flaviviridae, it is thus reasonable to assume that HPgV-2 may utilize the similar mechanism to independently control its fidelity. Further study about the activity and working mechanisms of HPgV-2 RdRp is ongoing. The significant difference of HPgV-2 and HCV makes HPgV-2 a new model to investigate RNA virus diversity and the mechanism of HPgV-2 polymerase in modulating virus replication.

In our study, we adapted the diversity rate of iSNVs and the analysis of co-occurring iSNVs to evaluate virus evolutionary dynamics. Our results revealed greater evolutionary rate of HCV HVR1 than the equivalent E1/2 gene of HPgV-2 and much less co-occurring iSNVs in HPgV-2 than HCV. All these results show the intrahost diversity of viral genome. The information of evolutionary dynamics is insufficient in the study. First, next-generation sequencing (NGS) relies on the *in silico* reconstruction of the viral genomes from mass short reads, and the assembly of whole viral genome sequence for single virus is very difficult and computationally extensive. Conventionally, evolutionary dynamics analysis uses molecular cloning of existing viral variants. The number of analyzed sequences may be not big enough. Low frequency variants and rare mutations could be largely ignored. Second, we only analyzed HCV HVR1 region or the HPgV-2 E2 gene fragment and not the whole HCV or HPgV-2 genome. The results may not represent the true evolutionary dynamics although the small high variable region is very informative and is thus suitable for the analysis of viral dynamics (Brown et al., 2005).

One major limit of our study is that we use iSNVs to represent the intrahost variation rather than the whole viral genomes. The results may not reflect the true genetic diversity of HPgV-2. In addition, due to the relatively short read-lengths generated by NGS, the assembly of whole viral genome sequence for single virus is very difficult (Sundquist et al., 2007); therefore, analysis of intrahost diversity can only apply to subgenomic fragments, such as HCV HVR1 or reverse transcriptase domain of HBV (Brambilla et al., 1998; Pawlotsky et al., 1998; Aragri et al., 2016). Furthermore, only a few HPgV-2 sequences are available in the public databases. More sequences worldwide are needed to investigate genetic diversity and to trace the evolution of HPgV-2 due to the interhost difference. In addition, the results about the immune responses caused by HPgV-2 and their role in regulating HPgV-2 replication are quite preliminary and insufficient since it is quite difficult to find HPgV-2 mono-infection. The cytokine expression profile observed in one patient before and after HCV elimination could only provide clues about differential cytokine expression between HPgV-2/HCV coinfection and HPgV-2 mono-infection but could not provide sufficient evidence to demonstrate the difference of immune responses between HPgV-2/HCV coinfection and HPgV-2 mono-infection. Moreover, HPgV-2 mono-infection is rarely identified (Wang et al., 2018b). It is thus difficult to find appropriate samples to make a direct comparison of immune response between HPgV-2 mono-infected patients and healthy individuals. Furthermore, we could not get the data about iSNVs for HPgV-2 mono-infection. However, we have analyzed the profile of differentially expressed cytokine between HPgV-2/HCV-coinfected patients with HCV elimination by DAA therapy and healthy blood donor.

In conclusion, this study provided new insights into the intrahost genomic variations and evolutionary dynamics of HPgV-2. Our preliminary results indicate that low immune selection pressure may be responsible for the relatively moderate genetic diversity of HPgV-2. Further study about the mechanisms of HPgV-2 RdRp in modulating virus replication fidelity is needed.

## Data Availability Statement

The datasets presented in this study can be found in online repositories. The names of the repository/repositories and accession number(s) can be found below: NCBI under accession PRJNA743193.

## Ethics Statement

The studies involving human participants were reviewed and approved by This study has been approved by the ethical committee of Nanfang Hospital (NFEC-2017-046) and Guangzhou Eighth People’s Hospital (No.201816107) according to the Declaration of Helsinki. The patients/participants provided their written informed consent to participate in this study.

## Author Contributions

ST and YL conceived and designed the experiments. FH, HF, LL, YZ, and WC collected the clinical sample and data. YL, ZW, HW, and JS performed the experiments. YL, HF, YT, and ST interpreted the results. YL made the tables and figures. YL and ST wrote the paper. All authors reviewed, revised, and approved the final report. All authors contributed to the article and approved the submitted version.

## Funding

This study was supported by grants from the National Natural Science Foundation of China (grant numbers 31872641, 81772923) and the National Major Science and Technology Project (grant number 2017ZX10202101-003).

## Conflict of Interest

The authors declare that the research was conducted in the absence of any commercial or financial relationships that could be construed as a potential conflict of interest.

## Publisher’s Note

All claims expressed in this article are solely those of the authors and do not necessarily represent those of their affiliated organizations, or those of the publisher, the editors and the reviewers. Any product that may be evaluated in this article, or claim that may be made by its manufacturer, is not guaranteed or endorsed by the publisher.

## Supplementary Material

Supplementary Figure 1 iSNV distributions along HCV HVR1. **(A)** Total iSNVs numbers along HCV HVR1 in six samples from three HPgV-2/HCV-coinfected patients. **(B)** The #iSNVs/kb values at HCV HVR1. **(C)** Distribution of iSNVs at codon positions. **(D)** Distribution of nonsynonymous (N)/synonymous (S) iSNVs. **(E)** Proportion of nucleotide substitutions among all iSNV sites identified from three patients (The samples of first time point) are shown in pie chart. **(F)** Mean allele frequency of each nucleotide substitution identified from three patients (The samples of first time point), T>C indicated T change to C. **(G)** Box plots of mutated allele frequency (MuAF) for nonsynonymous and synonymous iSNVs of HCV HVR1. Boxes represent the interquartile range (IQR) between the first and third quartiles; horizontal lines inside the boxes indicate the median. Statistical tests were performed between nonsynonymous and synonymous iSNVs of each ORF. WRS test, Wilcoxon rank-sum test; KS test, Kolmogorov-Smirnov test.

Supplementary Figure 2 Pathway analysis of differentially expressed proteins between a HPgV-2/HCV-coinfected patient and HCV mono-infected patient **(A**, **B)** as well as between a HPgV-2 mono-infected patient and a healthy donor **(C**, **D)**.**(A)** The Gene Ontology processes of the 32 differentially expressed cytokines between a HPgV-2/HCV-coinfected patient and HCV mono-infected patient were analyzed and enriched by Metascape platform, and the top 13 terms are shown in bar chart. **(B)** The interaction networks of biological process using Cytoscape plug-in ClueGO show that the 32 differentially expressed cytokines are mainly enriched in the positive regulation of nitric-oxide synthase biosynthetic process, neutrophil chemotaxis, cell-cell adhesion mediator activity, and cytokine-cytokine receptor interaction. **(C)** The Gene Ontology processes of the 52 differentially expressed cytokines between a HPgV-2 mono-infected patient and a healthy donor were analyzed and enriched by Metascape platform, and the top 20 terms are shown in bar chart. **(D)** The interaction networks of biological process using Cytoscape plug-in ClueGO show that the 52 differentially expressed cytokines are mainly enriched in the regulation and interaction of cytokine and its receptor, positive regulation of interleukin-10 (IL-10) production, and growth factor receptor binding. Multiple color dots indicate that it revolved in multiple biological processes.
