# Supplementary figures and images for: Difference of Intrahost Dynamics of the Second Human Pegivirus and Hepatitis C Virus in HPgV-2/HCV-Coinfected Patients
Source: Front Cell Infect Microbiol. 2021 Aug 12;11:728415. doi: 10.3389/fcimb.2021.728415 (PMC8403064; doi:10.3389/fcimb.2021.728415)

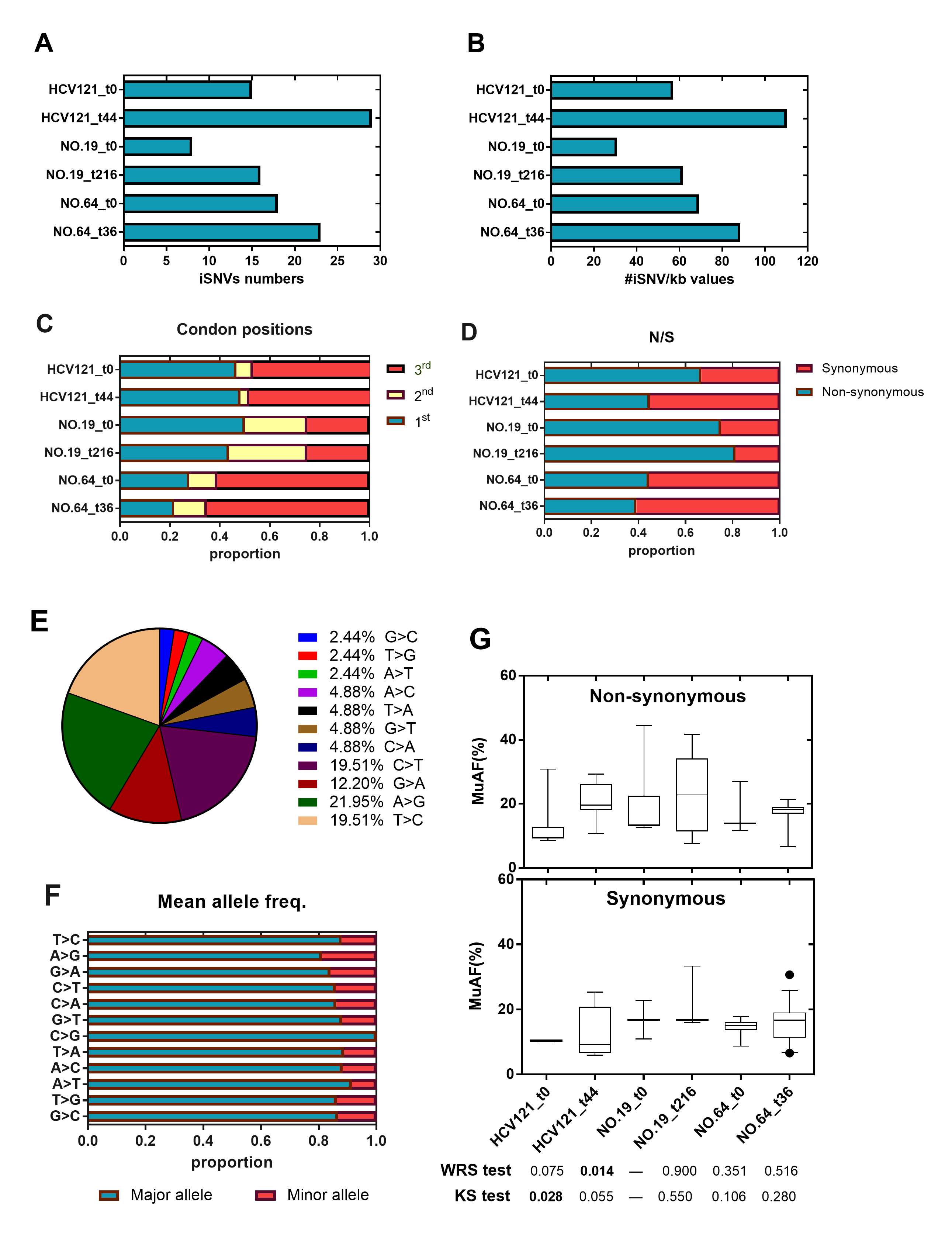

Supplement: Supplementary Figure 1 — iSNV distributions along HCV HVR1. (A) Total iSNVs numbers along HCV HVR1 in six samples from three HPgV-2/HCV-coinfected patients. (B) The #iSNVs/kb values at HCV HVR1. (C) Distribution of iSNVs at codon positions. (D) Distribution of nonsynonymous (N)/synonymous (S) iSNVs. (E) Proportion of nucleotide substitutions among all iSNV sites identified from three patients (The samples of first time point) are shown in pie chart. (F) Mean allele frequency of each nucleotide substitution identified from three patients (The samples of first time point), T>C indicated T change to C. (G) Box plots of mutated allele frequency (MuAF) for nonsynonymous and synonymous iSNVs of HCV HVR1. Boxes represent the interquartile range (IQR) between the first and third quartiles; horizontal lines inside the boxes indicate the median. Statistical tests were performed between nonsynonymous and synonymous iSNVs of each ORF. WRS test, Wilcoxon rank-sum test; KS test, Kolmogorov-Smirnov test. [file Image_1.tif]

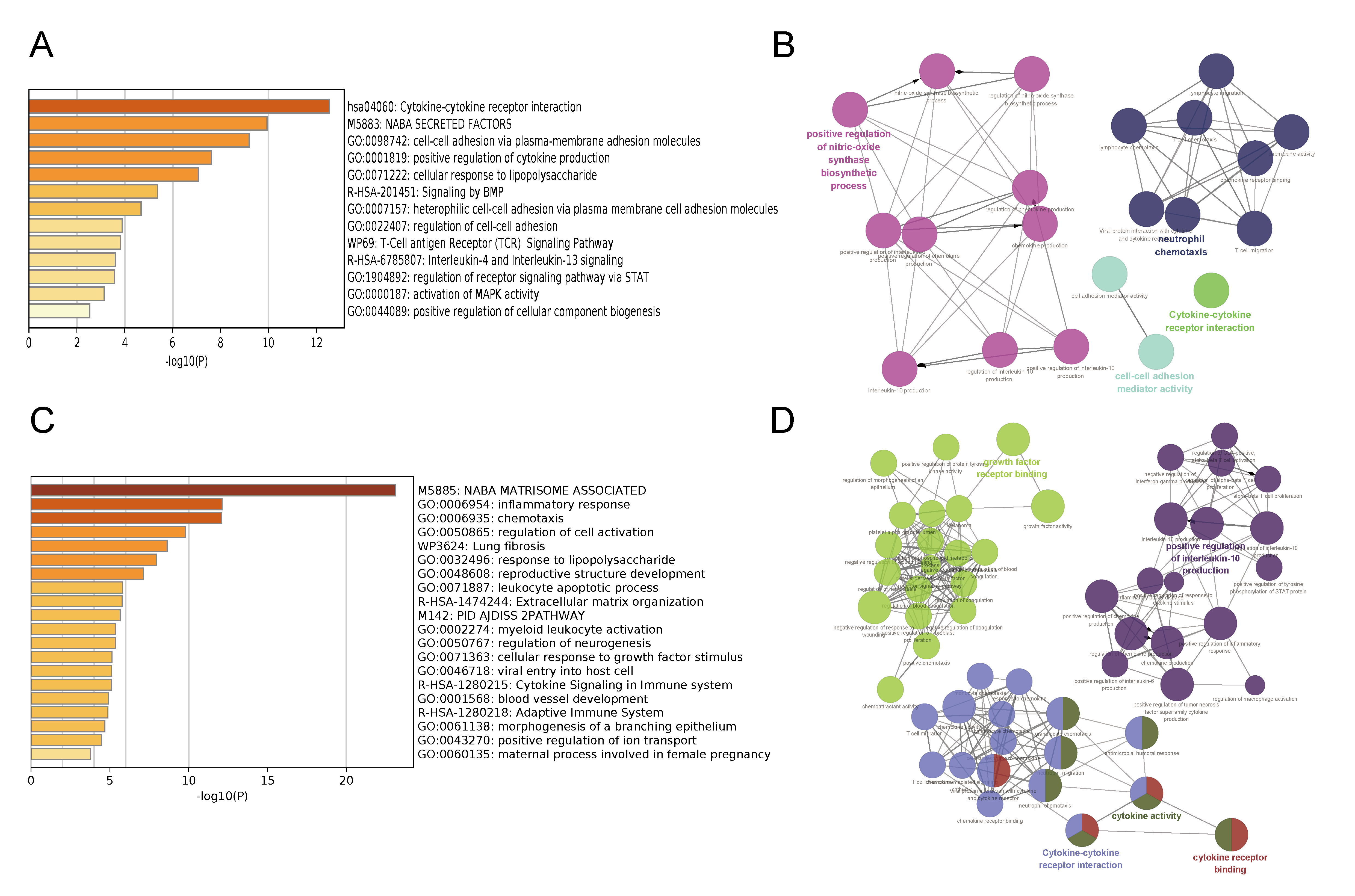

Supplement: Supplementary Figure 2 — Pathway analysis of differentially expressed proteins between a HPgV-2/HCV-coinfected patient and HCV mono-infected patient (A, B) as well as between a HPgV-2 mono-infected patient and a healthy donor (C, D).(A) The Gene Ontology processes of the 32 differentially expressed cytokines between a HPgV-2/HCV-coinfected patient and HCV mono-infected patient were analyzed and enriched by Metascape platform, and the top 13 terms are shown in bar chart. (B) The interaction networks of biological process using Cytoscape plug-in ClueGO show that the 32 differentially expressed cytokines are mainly enriched in the positive regulation of nitric-oxide synthase biosynthetic process, neutrophil chemotaxis, cell-cell adhesion mediator activity, and cytokine-cytokine receptor interaction. (C) The Gene Ontology processes of the 52 differentially expressed cytokines between a HPgV-2 mono-infected patient and a healthy donor were analyzed and enriched by Metascape platform, and the top 20 terms are shown in bar chart. (D) The interaction networks of biological process using Cytoscape plug-in ClueGO show that the 52 differentially expressed cytokines are mainly enriched in the regulation and interaction of cytokine and its receptor, positive regulation of interleukin-10 (IL-10) production, and growth factor receptor binding. Multiple color dots indicate that it revolved in multiple biological processes. [file Image_2.tif]
